# Charge Transfer-Mediated Dramatic Enhancement of Raman Scattering upon Molecular Point Contact Formation

**DOI:** 10.1021/acs.nanolett.1c02626

**Published:** 2022-02-21

**Authors:** Borja Cirera, Yair Litman, Chenfang Lin, Alaa Akkoush, Adnan Hammud, Martin Wolf, Mariana Rossi, Takashi Kumagai

**Affiliations:** † Department of Physical Chemistry, Fritz-Haber Institute of the Max-Planck Society, Faradayweg 4-6, 14195 Berlin, Germany; ‡ MPI for Structure and Dynamics of Matter, Luruper Chaussee 149, 22761 Hamburg, Germany; § Department of Inorganic Chemistry, Fritz-Haber Institute of the Max-Planck Society, Faradayweg 4-6, 14195 Berlin, Germany; ∥ Center for Mesoscopic Sciences, Institute for Molecular Science, Okazaki 444-8585, Japan

**Keywords:** Tip-enhanced Raman spectroscopy, Single-molecule spectroscopy, Current-carrying molecular Junction, Plasmonic nanocavity

## Abstract

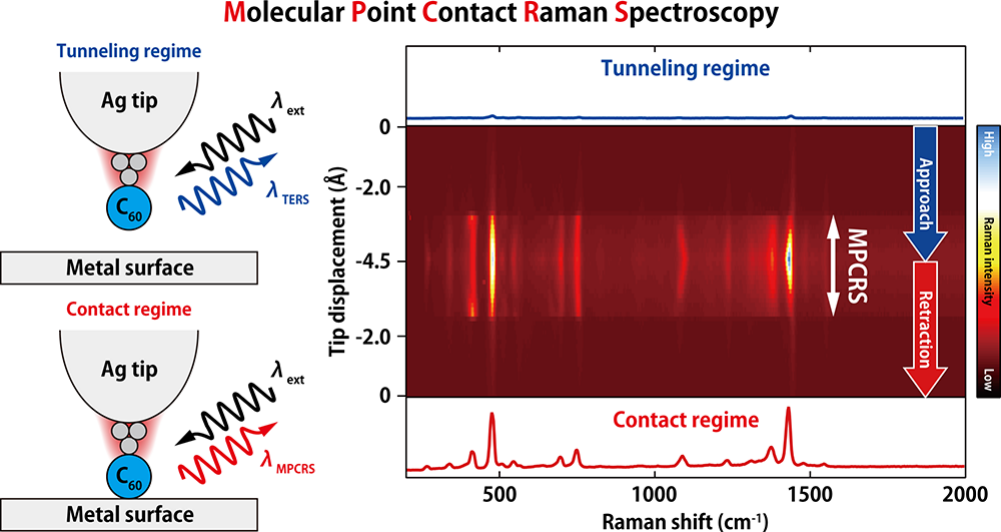

Charge-transfer enhancement of Raman scattering plays a crucial role in current-carrying molecular junctions. However, the microscopic mechanism of light scattering in such nonequilibrium systems is still imperfectly understood. Here, using low-temperature tip-enhanced Raman spectroscopy (TERS), we investigate how Raman scattering evolves as a function of the gap distance in the single C_60_-molecule junction consisting of an Ag tip and various metal surfaces. Precise gap-distance control allows the examination of two distinct transport regimes, namely tunneling regime and molecular point contact (MPC). Simultaneous measurement of TERS and the electric current in scanning tunneling microscopy shows that the MPC formation results in dramatic Raman enhancement that enables one to observe the vibrations undetectable in the tunneling regime. This enhancement is found to commonly occur not only for coinage but also transition metal substrates. We suggest that the characteristic enhancement upon the MPC formation is rationalized by charge-transfer excitation.

Giant enhancement of Raman scattering using plasmonic nanostructures has attracted increasing interest because of its potential for ultrasensitive chemical analysis, known as surface- and tip-enhanced Raman scattering/spectroscopy (SERS and TERS).^1^ In particular, single-molecule SERS/TERS is a powerful tool to study molecular systems in nanoscale environments. Remarkably, advanced low-temperature TERS experiments recently demonstrated Raman imaging with the submolecular spatial resolution reaching ∼1.5 Å, enabling to visualize individual vibration modes in real space.^2,3^ The exceptional sensitivity of TERS can be obtained when a plasmonic tip is brought in close proximity to the adsorbed molecule anchored on a flat metal surface (below a few Å gap distance). In such extreme junctions, atomic-scale structures (corrugation) on metal nanostructures play a crucial role to generate atomically confined electromagnetic fields through excitation of localized surface plasmon resonance (LSPR).^4−6^ Also, quantum mechanical effects, for example, electron tunneling across the junction, have a significant impact on the gap plasmon,^7^ which will be related to the enhancement mechanisms in TERS. In addition to the electromagnetic enhancement effect through the LSPR excitation, chemical interactions between molecule and metal cluster(s) can also largely contribute to the Raman scattering enhancement.^8^ This chemical enhancement effect was found to be particularly important when the molecule is fused between two metal nanoclusters^,^^9^ which may be manifested as a dramatic change of SERS/TERS spectra in plasmonic nanojunction fused with molecules.^10,11^ In addition, a correlation between electric current (conductance) and Raman spectra of molecular junctions was also reported in SERS of mechanical break junction^12,13^ and “fishing-mode” TERS^14^ experiments, which is accounted for by molecular orientation in the junction. However, the exact mechanism is still imperfectly understood. More recently, we found that atomic-point contact formation in plasmonic scanning tunneling microscope (STM) junctions results in dramatic Raman enhancement, and the exceptional sensitivity is demonstrated for an ultrathin oxide film on the Ag(111) surface^15^ and even for a Si(111)-7 × 7 surface.^16^ Here, we show that the dramatic Raman enhancement is operative also for molecular point contact (MPC) using single C_60_ junctions and propose that the underlying mechanism is rationalized by charge transfer enhancement.

We first show TERS of C_60_ molecules adsorbed on the Ag(111) surface. Figure 1a depicts schematically the TERS experiment in the STM junction consisting of an Ag tip, an ordered monolayer of C_60_ molecules, and the Ag(111) surface kept at ∼10 K (see Supporting Information for details). The junction is illuminated by a narrowband continuous-wave laser at a wavelength (λ_ext_) of 532 or 633 nm, which generates a tightly confined field at atomic-scale protrusions existing on the tip apex.^17^Figure 1b shows the STM image of C_60_ islands on the Ag(111) surface recorded under illumination (λ_ext_ = 532 nm) with an incident power density (*P*_inc_) of 0.33 mW cm^–2^ at the junction. The STM appearance represents the lowest unoccupied molecular orbital (LUMO) when a hexagon of C_60_ is facing toward the surface,^18^ which is in agreement with previous simulations.^19^ The stationary tripod shape also indicates the absence of rotation of the C_60_ molecules. Although no far-field Raman signal is detected, the intense Raman peaks from the C_60_ molecules can be observed when the Ag tip is brought into the tunneling regime (Figure 1c). The Raman intensity (*I*_Raman_) linearly depends on the *P*_inc_ (see Figure S1 in Supporting Information), indicating a spontaneous Raman process. The *I*_Raman_ is affected by the tip conditions, whereas the peak positions are not significantly shifted (see Figure S2 in Supporting Information). We estimated the spatial resolution to be <1 nm by recording TERS at the edge of a C_60_ island (Figure 1d,e).

**Figure 1:**
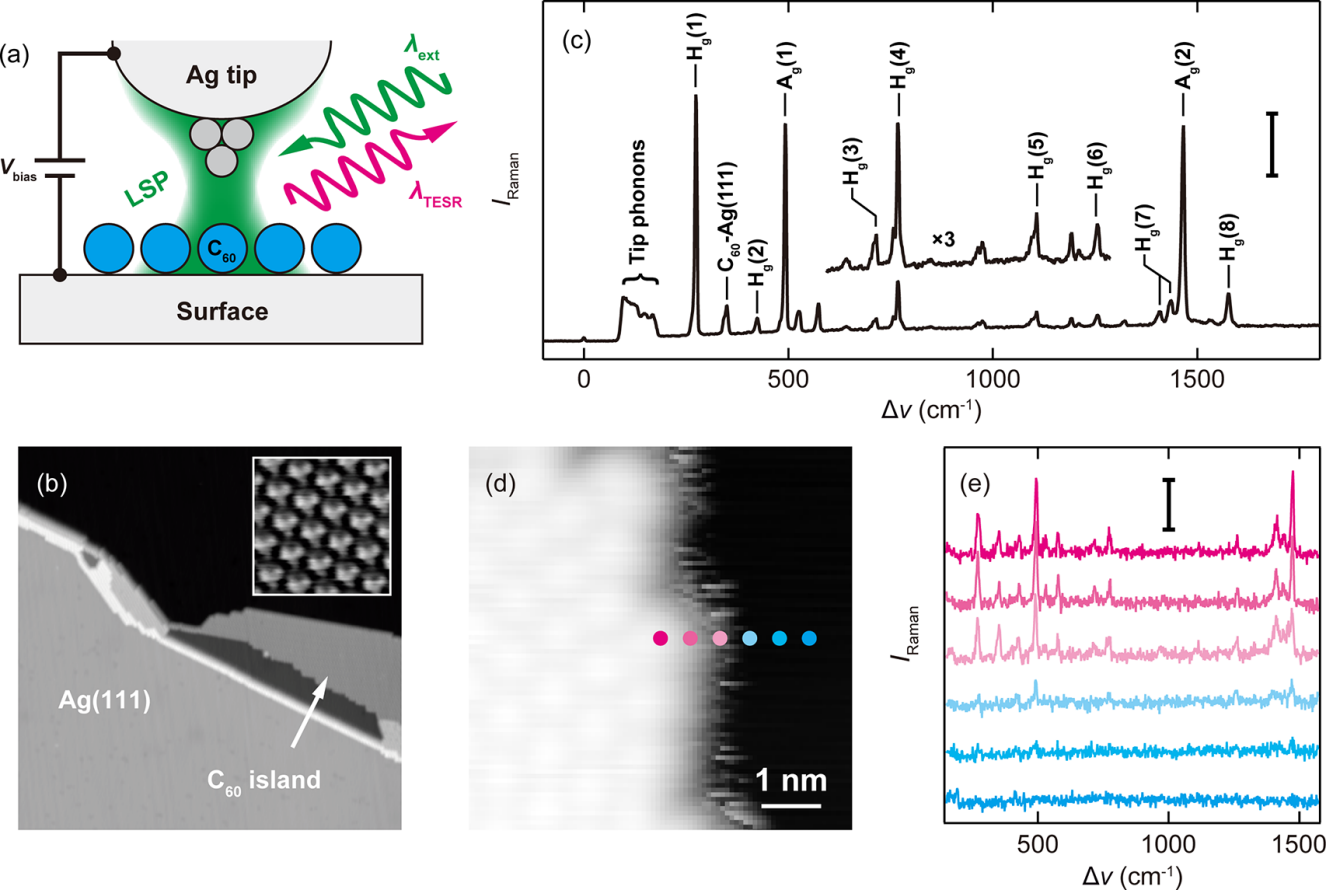
(a) Scheme of the TERS experiment. (b) STM image of C_60_ molecules on Ag(111) under illumination at λ_ext_ = 532 nm at 10 K (*V*_bias_ = 0.6 V, *j*_STM_ = 100 pA; inset: *V*_bias_= 0.1 V, *j*_STM_ = 2.6 nA). (c) TERS spectra obtained over a C_60_ molecule in the island at λ_ext_ = 532 nm (Ag tip, λ_ext_ = 532 nm, *P*_inc_ = 0.33 mWμm^–2^, 10 K, scale bar = 500 cps). (d) STM image of the edge of a C_60_ island where the Raman spectra of (e) are acquired (*V*_bias_ = 10 mV, *j*_STM_ = 10 pA). (e) TERS spectra acquired across the edge of the island. The location is indicated in (d) (*V*_bias_ = 10 mV, *j*_STM_ = 10 pA, λ_ext_ = 633 nm, *P*_inc_ = 0.5 × 10^5^ W cm^–2^, 10 K, scale bar = 500 cps). All acquisition parameters are listed in Table S1.

The TERS peaks of C_60_ are assigned according to previous Raman studies of a solid-state sample at 20 K and the isolated C_60_ molecule^20,21^ as well as the DFT simulations conducted for the experimental configuration (see Figure S3 in Supporting Information). The calculated frequencies of the Raman active C_60_ vibrations on Ag(111) and in the gas phase are listed in Table S2 (Supporting Information). An isolated C_60_ molecule has in total 174 vibrational degrees of freedom and the icosahedral symmetry yields 10 distinct Raman-active modes (2*A*_g_ + 8*H*_g_). The TERS spectrum involves all of the Raman active modes, and the relative intensities between the *A*_g_ and *H*_g_ modes are also similar to those observed in a solid state for λ_ext_ = 514 nm,^22^ whereas most of the modes are red-shifted compared to those in a solid state. Red-shifts of C_60_ vibrations were also observed in SERS on a rough Ag substrate.^23^ The observed red-shifts on the Ag(111) surface are confirmed by the DFT simulations (see Table S2 in Supporting Information), which can be attributed to softening of the C_60_ modes due to the electronic density rearrangement through orbital hybridization between C_60_ and Ag(111). As can be seen in Figures S4 and S5 (Supporting Information), the unoccupied molecular states strongly hybridize with the surface, and the LUMO is partially filled. This may be the origin of the vibrational red-shifts because electron transfer to an antibonding orbital delocalized over the entire molecule causes expansion of the molecule and hence softening of the intramolecular bonds.

The TERS peaks in Figure 1c have a shoulder (the *H*_g_(7) mode appears to be split). This could arise from lifting of vibrational degeneracies for the *H*_g_ modes due to contact with the surface (see Table S3 in Supporting Information). However, because the *A*_g_ modes are not degenerate, the shoulder might involve interference between the electronic and vibrational Raman scattering pathways, yielding a Fano-like line shape.^24^ Furthermore, the TERS spectrum shows more vibrational modes in addition to the 10 Raman-active modes of free C_60_. The peak at 347 cm^–1^ was observed in the previous SERS experiment,^23^ which can be assigned to the C_60_-surface “bouncing” mode based on the DFT simulations. The other peaks that are Raman nonactive in the isolated C_60_ molecule appear due to symmetry-lowering caused by the adsorption onto the surface.

Next, we examine the gap-distance dependence of single-C_60_ TERS including reversible formation and breaking of MPC. As depicted in Figure 2, a single C_60_ molecule on Ag(111) is transferred to the Ag tip apex (hereafter denoted as C_60_-tip), and then it is moved toward the bare Ag(111) surface until the molecule contacts the surface and subsequently it is retracted. The middle panel of Figure 2c displays a waterfall plot of the TERS spectra recorded as a function of relative displacement of the tip–surface distance (Δ*z*) when the C_60_-tip approaches the surface. The vertical and horizontal axis corresponds to Δ*z* and Raman shift (Δν), respectively, and the color scale represents *I*_Raman_. A remarkable observation is the abrupt increase of the TERS intensity when the C_60_-tip contacts the surface. The MPC formation is evident in the STM current (*j*_STM_) simultaneously recorded with the TERS spectra. The *j*_STM_ shows a well-known jump-to-contact behavior that occurs when the junction is fused by a point contact.^25^ The symmetric behavior of the TERS spectra and the *j*_STM_–Δ*z* curve with respect to the turning point indicates that the process is reversible. The TERS intensity is not dependent on the amount of the direct current flowing in the junction (see Figure S6 in Supporting Information).

**Figure 2:**
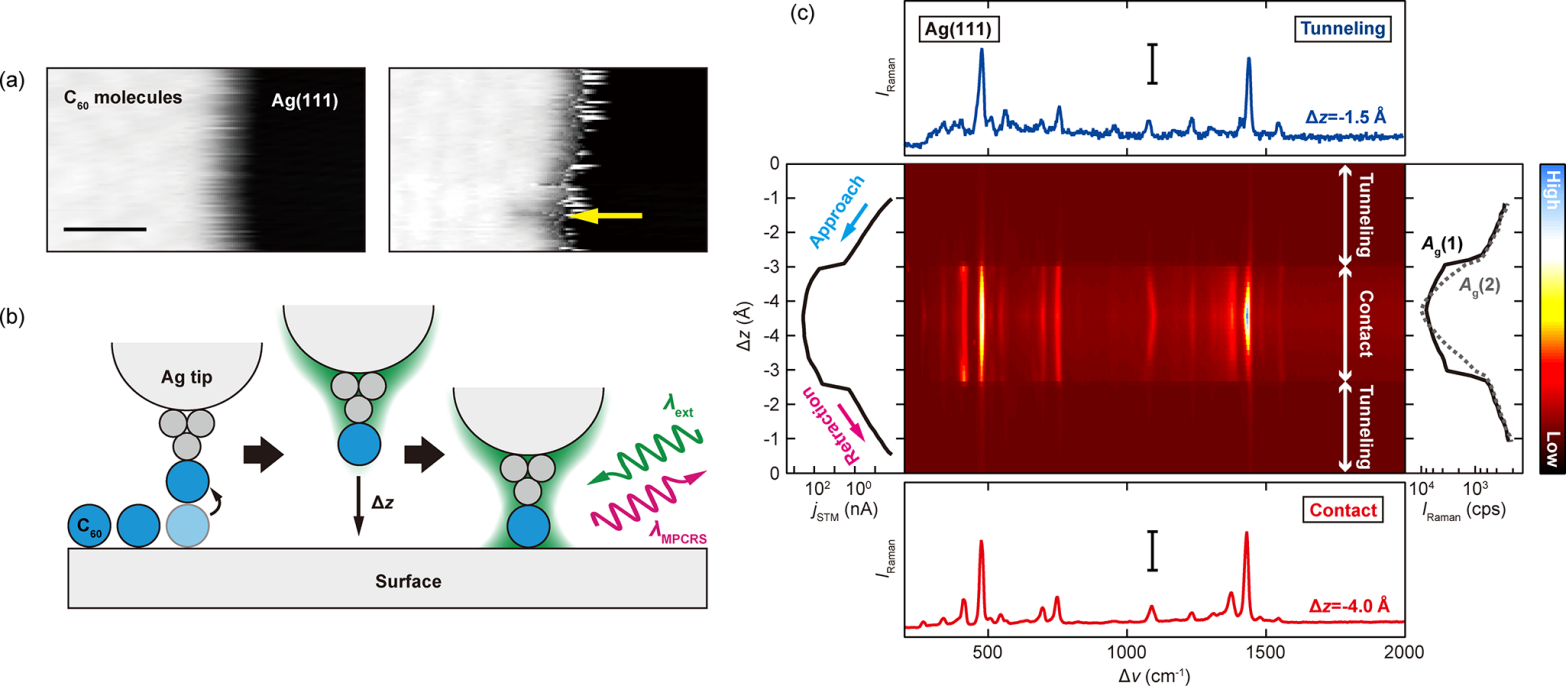
(a) STM images before and after picking a single C_60_ molecule from the island (*V*_bias_ = 0.5 V, *j*_STM_ = 50 pA, 10 K, scale bar = 2 nm). (b) Schematic of the Δ*z*-dependent TERS measurement in a single C_60_ molecule junction. (c) Δ*z*-dependent TERS spectra measured on the Ag(111) surface recording one cycle of C_60_-tip approach and retraction (λ_ext_ = 532 nm, *P*_inc_ = 0.33 mWμm^–2^, 10 K). The left panel shows the simultaneously obtained *j*_STM_–Δ*z* curve. Although the *V*_bias_ is nominally set to zero, the *j*_STM_ occurs due to a photovoltage (estimated to be ∼1 mV). The right panel shows the intensity of the *A*_g_ modes as a function of the Δ*z*. The color scale correspods to 600–12000 cps. The top and bottom panels show the TERS spectra in the tunneling and MPC regime, respectively. The scale bar corresponds to 200 (top) and 5000 cps (bottom).

In Figure 2c, some of the vibration modes exhibit a continuous peak shift as a function of Δ*z*, which is more pronounced after MPC formation. The DFT calculations predict that a mechanical deformation of C_60_ results in blue-shifts for all vibrational modes (see Table S4 in Supporting Information), whereas the electronic charge rearrangement caused by the MPC formation results in red-shifts as discussed above. In experiment, we observe that some vibrational peaks are red-shifted as the Δ*z* decreases (e.g. *H*_*g*_(7), *H*_*g*_(8), and *A*_*g*_(2), see Figure S7 in Supporting Information), while some peaks are blue-shifted (e.g., *H*_*g*_(5)) when the MPC is further squeezed, implicating complex contributions from the mechanical deformation and the charge density rearrangement.

In order to demonstrate that the MPC-induced Raman enhancement is not a peculiar phenomenon of the Ag tip–C_60_–Ag(111) junction, we performed the same experiment on the Au(111), Cu(111), and Pt(111) surfaces (Figure 3a–c, respectively). On Au(111), the TERS intensity in the tunneling regime becomes smaller than that on Ag(111) due to the reduced field enhancement compared to Ag. The Cu(111) surface interacts with C_60_ more strongly than Ag(111) and Au(111), while the plasmonic enhancement is expected to be similar to Au in the visible range. In addition, as an example of transition metals, we used the Pt(111) surface that is not generally used in TERS due to its weaker plasmonic resonance in the visible regime compared to coinage metals.^26^ Indeed, the TERS intensity on Pt(111) is very weak in the tunneling regime (Figure 3c). However, for all these surfaces the intense Raman signals appear abruptly upon MPC formation. We evaluated the enhancement factor ρ_MPC_ for the *A*_g_(2) peak on each substrate, which is defined as the ratio between the intensity at 1 Å above and below the MPC: ρ_MPC,Ag(111)_ = 15.4 ± 0.4, ρ_MPC,Au(111)_ = 275 ± 15, ρ_MPC,Cu(111)_ = 29.3 ± 2.6, ρ_MPC,Pt(111)_ = 78.8 ± 6.4. The exact enhancement factors are affected by the LSPR properties of the junction, the excitation wavelength, and possibly the adsorption geometry of C_60_ on the tip. However, these results indicate that the exceptional sensitivity of MPC-TERS can be commonly obtained for different metal substrates.

**Figure 3:**
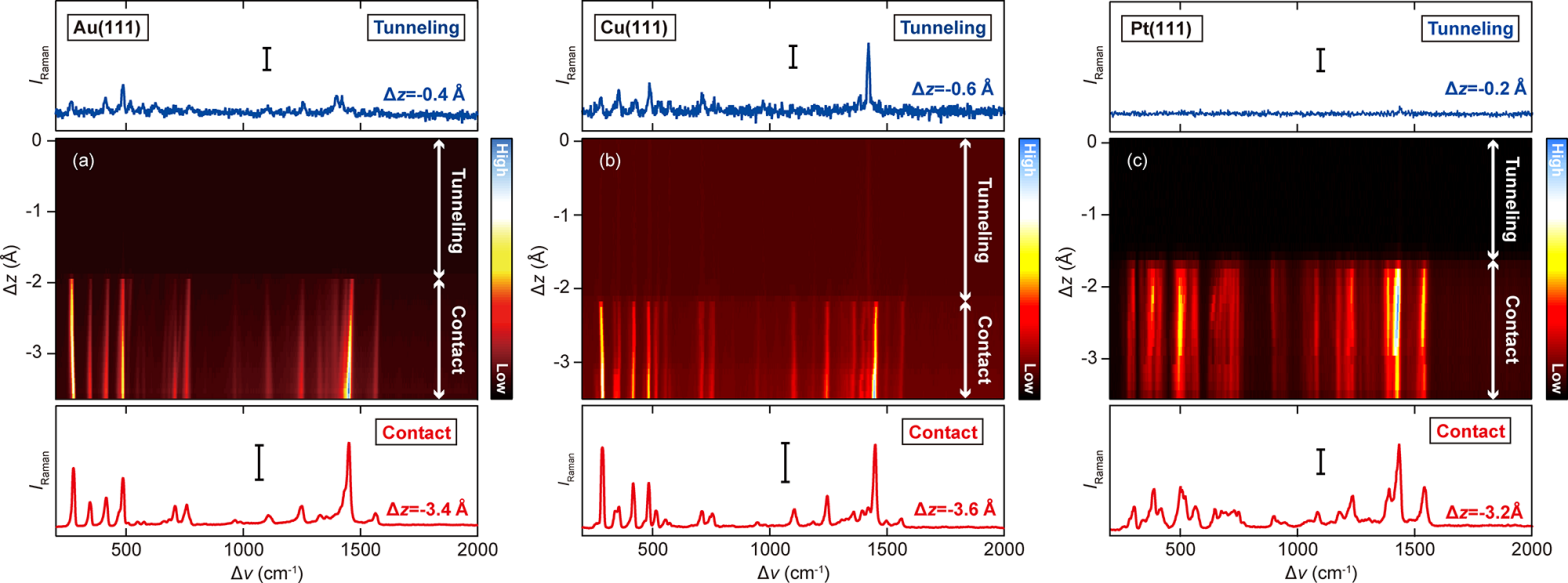
(a–c) Δ*z*-dependent TERS spectra measured on the Au(111), Cu(111), and Pt(111) surfaces, respectively (Au(111): λ_ext_ = 532 nm, *P*_inc_ = 0.33 mWμm^–2^, *V*_bias_ = 0 V, 10 K, Cu(111): λ_ext_ = 633 nm, *P*_inc_ = 0.45 mWμm^–2^, *V*_bias_ = 0 V, 10 K, Pt(111): λ_ext_ = 633 nm, *P*_inc_ = 0.45 mWμm^–2^, *V*_bias_ = 0 V, 10 K). The color scale corresponds to (a) 600–200 000 cps, (b) 0–16 000 cps, (c) 1500–5000 cps. The top and bottom panels show the TERS spectra in the tunneling and MPC regime, respectively. The scale bar corresponds to 100 (top) and (a) 10 000, (b) 5000, (c) 1000 cps (bottom).

In order to further examine the impact of the contact surface on the TERS enhancement at the MPC, we measured a double-C_60_ junction on Ag(111) (Figure 4a). As can be seen in Figure 4b, both *j*_STM_–Δ*z* curve and TERS intensity do not exhibit an abrupt change. The *j*_STM_–Δ*z* curve is in agreement with previous experiments on Cu(111).^27^ The absence of a jump-to-contact behavior in the contact regime is explained by a gradual transition of the interaction between two C_60_ molecules from the attractive (van der Waals) to repulsive (Pauli) range.^28^ This result shows that chemical interactions at the MPC play a critical role in the enhancement mechanism.

**Figure 4:**
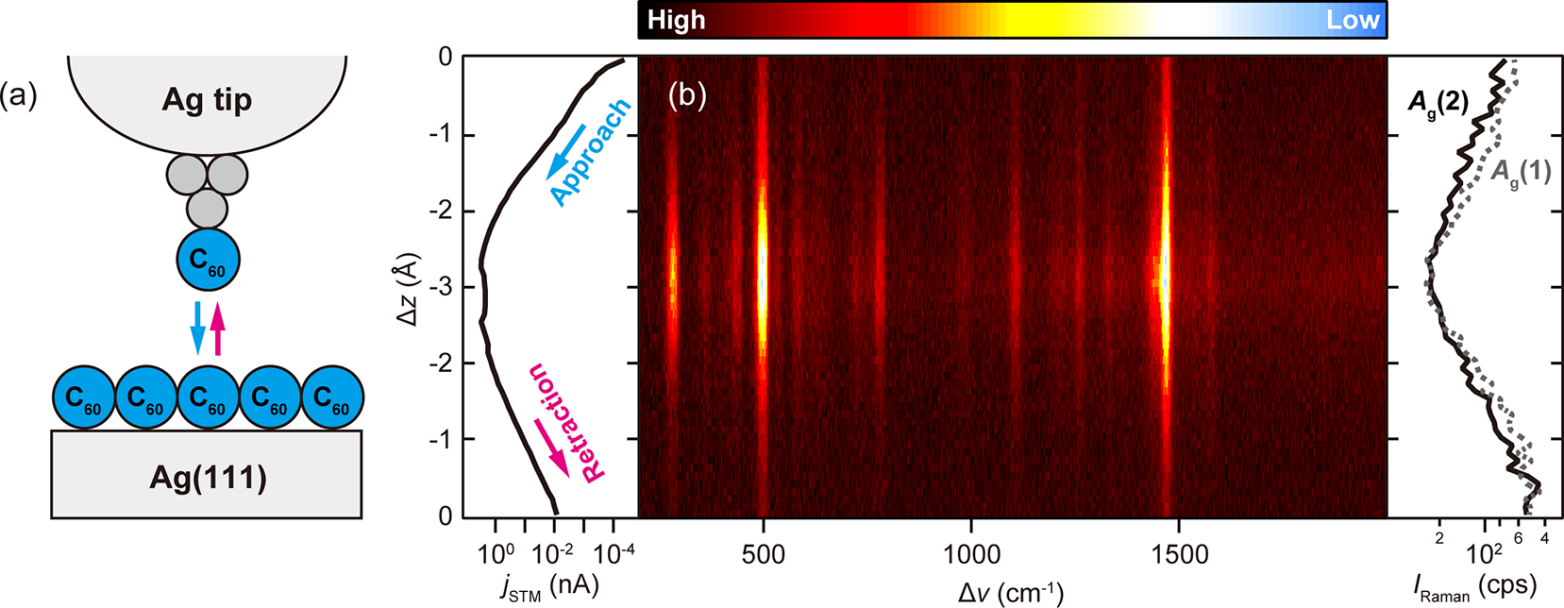
(a) Schematic of the Δ*z*-dependent TERS measurement in a C_60_–C_60_ junction. (b) Δ*z*-dependent TERS spectra obtained for one approach and retraction cycle of a monolayer C_60_ film on Ag(111) (λ_ext_ = 532 nm, *P*_inc_ = 0.33 mWμm^–2^, *V*_bias_ = 0 V, 10 K), together with the simultaneously obtained *j*_STM_–Δ*z* curve (left) and the intensity of the *A*_g_ modes (right). The color scale corresponds to 750–1100 cps.

The enhancement in SERS/TERS can be generally classified into electromagnetic (EM) and chemical effects. The former is determined by the plasmonic properties of metallic nanostructures. Theoretically, the chemical effects can be further classified into (1) chemical interactions (orbital hybridization) in a molecule–metal system at the electronic ground state, which changes the static polarizability, (2) charge transfer resonance including excited states of a hybrid molecule–metal system (CT), or (3) resonant transition within molecular orbitals (resonance Raman, RR).^8^ The continuous increase of the TERS intensity in the tunneling regime will be dominated by the EM enhancement.

The MPC-induced enhancement will be explained by chemical effects rather than EM enhancement because an abrupt increase of the gap plasmon is unlikely.^5,6,29^ We first considered a change in the static polarizability whose square is proportional to the Raman intensity. To this end, we simulated the static polarizability tensor using a generalized-gradient density functional approximation (see Section 9 in Supporting Information). Although the computed value of the *zz* component of the polarizability tensor depends on the tip–C_60_ geometry (see Table S5 in Supporting Information) and the magnitude of the lateral lattice vectors of the simulation cell (see Table S6 and Figure S8 in Supporting Information), its change before and after MPC formation does not rationalize the observed enhancement factors. Therefore, we believe that the abrupt Raman enhancement at the MPC is explained by an additional charge-transfer contribution. This mechanism is associated with the local electronic structure of the system. Scanning tunneling spectroscopy (STS) shows that a significant change of the local electronic structure occurs upon the MPC formation. As can be seen in Figure 5a, the STS intensity exhibits a peak around zero-bias at the MPC, which is absent in the tunneling regime and indicates the increase of the density of states (DOS) around the Fermi level. A significant change in the local DOS may be consistent with the DFT simulations (Figure 5b–d, see also Section 5 in Supporting Information). Figure 5c,d displays the calculated projected DOS for the C_60_-tip and the MPC. The C_60_ states at the MPC are further broadened than those for the C_60_-tip configuration. In the tunneling regime (Figure 5c), relatively narrow molecular states may lead to a strong wavelength dependence for the RR process. Similarly, resonant CT into the excited states may not be efficient because the transition is limited within the reach with the visible excitation. These processes will be largely affected upon the MPC formation (Figure 5d). The broadened molecular states may lead to additional RR and CT channels and the latter involves transition from the continuum states of both tip and surface to the molecular states (and *vice versa*).^30,31^ The MPC-induced enhancement occurs for a different excitation wavelength (see Figure S9 in Supporting Information), which may be consistent with widely spread resonant channels. The charge-transfer mechanism is also consistent with the result of the double-C_60_ junction because the change of the DOS is less pronounced due to the weak interaction between two molecules, which results in a reduced orbital hybridization in the junction and thus hampers the additional charge-transfer enhancement. The charge-transfer enhancement at the MPC will be generally operative for other metallic substrates as orbital hybridization and a concomitant change of the DOS upon MPC formation is commonly expected.^32,33^ Additionally, the chemical enhancement mechanism induced by charge transfer could be further modified if the applied *V*_bias_ results in the redistribution of the electron density within the molecule in the junction.^34,35^

**Figure 5:**
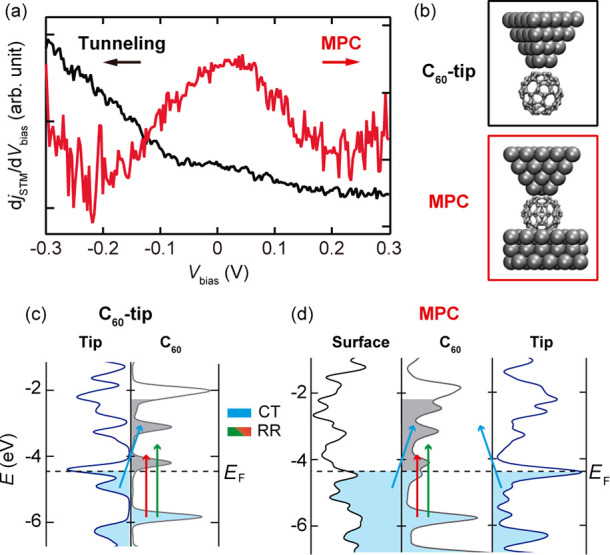
(a) Scanning tunneling spectra obtained for a C_60_-tip in the tunneling and MPC (black: set-point of *V*_bias_ = −300 mV, *j*_STM_ = 5 nA, *V*_mod_ = 5 mV at 883 Hz, red: set-point of *V*_bias_ = −300 mV, *j*_STM_ = 27 μA, *V*_mod_ = 5 mV at 883 Hz). (b) Models of C_60_-tip and MPC used in the DFT calculations. (c,d) Calculated projected density of states for C_60_-tip and MPC. The arrows show possible resonance paths in the system (blue arrow: CT, red/green arrows: molecular resonance). The gray areas represent the molecular unoccupied states that can be reached with λ_ext_ = 532 nm.

The selection rule with an extremely confined field is another important subject in TERS. However, significant mixing of the normal modes, caused by adsorption of C_60_ on the surface (and/or tip), hampers to clarify the symmetry of the vibration modes (see Table S3 in Supporting Information). Additionally, the detailed information on the field distribution in the junction is also not available. A strong local field-gradient might break the conventional selection rule that is based on the dipole approximation.^36^ In the present case, however, the quadrupole or magnetic dipole active modes are not clearly observed. The contribution of the local field gradient may not be significant for relatively large molecules physically adsorbed on flat surfaces.^37^ In order to discuss the accurate selection rule, it is desirable to perform extended atomistic first-principles calculations which can provide a consistent treatment of atomistic structures, orbital hybridization and charge density responses (polarizability) as well as propagation of the EM fields in a unified manner, like the Maxwell–time-dependent DFT scheme.^38^

In summary, we investigated TERS of current-carrying molecular junctions including a single C_60_, and how Raman scattering evolves as a function of the gap distance. The transition from the tunneling to MPC regime was continuously monitored by moving C_60_-tip toward various single-crystal metal surfaces. By recording simultaneously TERS and the electric current in STM, we showed that the abrupt Raman enhancement occurs when the MPC is formed. This enhancement is commonly observed for different substrates exhibiting distinct plasmonic properties and the interaction with C_60_, namely Ag(111), Au(111), Cu(111), and Pt(111). We deduced that the MPC-induced Raman enhancement is rationalized by the chemical effects. Among the three distinct chemical enhancement effects, the DFT calculations predicted that the electronic charge rearrangement at the ground state (i.e., change of the static Raman polarizability) cannot account for the observed enhancement factors. Therefore, we proposed that the characteristic enhancement at the MPC originates from additional charge-transfer and resonance Raman channels in the hybrid tip–C_60_–surface system caused by renormalization and broadening of the local electronic states. This mechanism was further corroborated by examining the double-C_60_ junction where the charge transfer enhancement is significantly reduced due to the weak chemical interaction between the molecules. The exceptional sensitivity of MPC-TERS may extend the possibility of TERS to investigate catalytic and electrode reactions on transition metal surfaces. Our approach will also pave the way for studying light–matter coupling in nonequilibrium quantum transport systems^39^ where Raman scattering can address fundamental physics in molecular optoelectronics^40^ and optomechanics.^41^

## Supporting Information Available

Materials and methods, incident laser power dependence of the TERS intensity in the tunneling regime, tip condition dependence of TERS for a C_60_ molecule on Ag(111), models used in the DFT calculations, projected electronic density of states, detailed analysis of the Raman active modes of C_60_ on Ag(111) and C_60_-tip, Δ*z*-dependent TERS of a C_60_-tip on Ag(111) at small *V*_bias_, enlarged Δ*z*-dependent TERS of a C_60_-tip on Ag(111) highlighting the shifting of the vibrational modes, simulations of the static Raman polarizability of C_60_, wavelength dependence of MPC-TERS (PDF)

## References

[ref1] Zrimsek A. B.; Chiang N.; Mattei M.; Zaleski S.; McAnally M. O.; Chapman C. T.; Henry A.-I.; Schatz G. C.; Van Duyne R. P. Single-Molecule Chemistry with Surface- and Tip-Enhanced Raman Spectroscopy. Chem. Rev. 2017, 117 (11), 7583–7613. 10.1021/acs.chemrev.6b00552. 28610424

[ref2] Lee J.; Crampton K. T.; Tallarida N.; Apkarian V. A. Visualizing vibrational normal modes of a single molecule with atomically confined light. Nature 2019, 568, 78–82. 10.1038/s41586-019-1059-9. 30944493

[ref3] Zhang Y.; Yang B.; Ghafoor A.; Zhang Y.; Zhang Y.-F.; Wang R.-P.; Yang J.-L.; Luo Y.; Dong Z.-C.; Hou J. G. Visually Constructing the Chemical Structure of a Single Molecule by Scanning Raman Picoscopy. Natl. Sci. Rev. 2019, 6 (6), 1169–1175. 10.1093/nsr/nwz180. 34691995 PMC8291412

[ref4] Benz F.; Schmidt M. K.; Dreismann A.; Chikkaraddy R.; Zhang Y.; Demetriadou A.; Carnegie C.; Ohadi H.; de Nijs B.; Esteban R.; Aizpurua J.; Baumberg J. J. Single-molecule optomechanics in “picocavities. Science 2016, 354, 726–729. 10.1126/science.aah5243. 27846600

[ref5] Zhang P.; Feist J.; Rubio A.; García-González P.; García-Vidal F. J. *Ab initio* nanoplasmonics: The impact of atomic structure. Phys. Rev. B 2014, 90 (16), 161407 10.1103/PhysRevB.90.161407.

[ref6] Urbieta M.; Barbry M.; Zhang Y.; Koval P.; Sánchez-Portal D.; Zabala N.; Aizpurua J. Atomic-Scale Lightning Rod Effect in Plasmonic Picocavities: A Classical View to a Quantum Effect. ACS Nano 2018, 12 (1), 585–595. 10.1021/acsnano.7b07401. 29298379

[ref7] Zhu W.; Esteban R.; Borisov Ag. G.; Baumberg J. J.; Nordlander P.; Lezec H. J.; Aizpurua J.; Crozier K. B. Quantum mechanical effects in plasmonic structures with subnanometre gaps. Nat. Commun. 2016, 7, 11495 10.1038/ncomms11495. 27255556 PMC4895716

[ref8] Jensen L.; Aikens C. M.; Schatz G. C. Electronic structure methods for studying surface-enhanced Raman scattering. Chem. Soc. Rev. 2008, 37, 1061–1073. 10.1039/b706023h. 18443690

[ref9] Zhao L. L.; Jensen L.; Schatz G. C. Surface-Enhanced Raman Scattering of Pyrazine at the Junction between Two Ag_20_ Nanoclusters. Nano Lett. 2006, 6 (6), 1229–1234. 10.1021/nl0607378. 16771585

[ref10] Banik M.; Apkarian V. A.; Park T.-H.; Galperin M. Raman Staircase in Charge Transfer SERS at the Junction of Fusing Nanospheres. J. Phys. Chem. Lett. 2013, 4 (1), 88–92. 10.1021/jz3018072. 26291217

[ref11] El-Khoury P. Z.; Hu D.; Apkarian V. A.; Hess W. P. Raman Scattering at Plasmonic Junctions Shorted by Conductive Molecular Bridges. Nano Lett. 2013, 13 (4), 1858 10.1021/nl400733r. 23534898

[ref12] Ward D. R.; Halas N. J.; Ciszek J. W.; Tour J. M.; Wu Y.; Nordlander P.; Natelson D. Simultaneous Measurements of Electronic Conduction and Raman Response in Molecular Junctions. Nano Lett. 2008, 8 (3), 919–924. 10.1021/nl073346h. 18237152

[ref13] Konishi T.; Kiguchi M.; Takase M.; Nagasawa F.; Nabika H.; Ikeda K.; Uosaki K.; Ueno K.; Misawa H.; Murakoshi K. Single Molecule Dynamics at a Mechanically Controllable Break Junction in Solution at Room Temperature. J. Am. Chem. Soc. 2013, 135 (3), 1009–1014. 10.1021/ja307821u. 23072537

[ref14] Liu Z.; Ding S.-Y.; Chen Z.-B.; Wang X.; Tian J.-H.; Anema J. R.; Zhou X.-S.; Wu D.-Y.; Mao B.-W.; Xu X.; Ren B.; Tian Z.-Q. Revealing the molecular structure of single-molecule junctions in different conductance states by fishing-mode tip-enhanced Raman spectroscopy. Nat. Commun. 2011, 2, 305 10.1038/ncomms1310. 21556059 PMC3112534

[ref15] Liu S.; Cirera B.; Sun Y.; Hamada I.; Müller M.; Hammud A.; Wolf M.; Kumagai T. Dramatic Enhancement of Tip-Enhanced Raman Scattering Mediated by Atomic Point Contact Formation. Nano Lett. 2020, 20 (8), 5879–5884. 10.1021/acs.nanolett.0c01791. 32678605 PMC7458471

[ref16] Liu S.; Hammud A.; Wolf M.; Kumagai T. Atomic Point Contact Raman Spectroscopy of a Si(111)-7 × 7 Surface. Nano Lett. 2021, 21 (9), 4057–4061. 10.1021/acs.nanolett.1c00998. 33934600 PMC8288640

[ref17] Trautmann S.; Aizpurua J.; Götz I.; Undisz A.; Dellith J.; Schneidewind H.; Rettenmayr M.; Deckert V. A classical description of subnanometer resolution by atomic features in metallic structures. Nanoscale 2017, 9, 391–401. 10.1039/C6NR07560F. 27924333

[ref18] Franke K. J.; Pascual J. I. Effects of electron–vibration coupling in transport through single molecules. J. Phys.: Condens. Matter 2012, 24, 394002 10.1088/0953-8984/24/39/394002. 22964796

[ref19] Li H. I.; Pussi K.; Hanna K. J.; Wang L.-L.; Johnson D. D.; Cheng H.-P.; Shin H.; Curtarolo S.; Moritz W.; Smerdon J. A.; McGrath R.; Diehl R. D. Surface Geometry of C_60_ on Ag(111). Phys. Rev. Lett. 2009, 103 (5), 056101 10.1103/PhysRevLett.103.056101. 19792515

[ref20] Dong Z.-H.; Zhou P.; Holden J. M.; Eklund P. C.; Dresselhaus M. S.; Dresselhaus G. Observation of higher-order Raman modes in C_60_ films. Phys. Rev. B 1993, 48 (4), 2862–2865. 10.1103/PhysRevB.48.2862. 10008701

[ref21] Jishi R. A.; Mirie R. M.; Dresselhaus M. S. Force-constant model for the vibrational modes in C_60_. Phys. Rev. B 1992, 45 (23), 13685–13689. 10.1103/PhysRevB.45.13685. 10001462

[ref22] van Loosdrecht P. H. M.; van Bentum P. J. M.; Verheijen M. A.; Meijer G. Raman scattering in single crystal C_60_. Chem. Phys. Lett. 1992, 198 (6), 587–595. 10.1016/0009-2614(92)85034-8. 10046099

[ref23] Rosenberg A.; DiLella D. P. Anomalously enhanced Raman scattering from C_60_ on Ag surfaces. Chem. Phys. Lett. 1994, 223 (1–2), 76–81. 10.1016/0009-2614(94)00414-5.

[ref24] Dey S.; Banik M.; Hulkko E.; Rodriguez K.; Apkarian V. A.; Galperin M.; Nitzan A. Observation and analysis of Fano-like lineshapes in the Raman spectra of molecules adsorbed at metal interfaces. Phys. Rev. B 2016, 93 (3), 035411 10.1103/PhysRevB.93.035411.

[ref25] Kröger J.; Ńeel N.; Limot L. Contact to single atoms and molecules with the tip of a scanning tunnelling microscope. J. Phys.: Condens. Matter 2008, 20, 223001 10.1088/0953-8984/20/22/223001.

[ref26] Liu H. W.; Nishitani R.; Han T. Z.; Ie Y.; Aso Y.; Iwasaki H. STM fluorescence of porphyrin enhanced by a strong plasmonic field and its nanoscale confinement in an STM cavity. Phys. Rev. B 2009, 79 (12), 125415 10.1103/PhysRevB.79.125415.

[ref27] Schull G.; Frederiksen T.; Brandbyge M.; Berndt R. Passing Current through Touching Molecules. Phys. Rev. Lett. 2009, 103 (20), 206803 10.1103/PhysRevLett.103.206803. 20365999

[ref28] Brand J.; Néel N.; Kröger J. Probing relaxations of atomic-scale junctions in the Pauli repulsion range. New J. Phys. 2019, 21, 103041 10.1088/1367-2630/ab4c84.

[ref29] Savage K. J.; Hawkeye M. M.; Esteban R.; Borisov A. G.; Aizpurua J.; Baumberg J. J. Revealing the quantum regime in tunnelling plasmonics. Nature 2012, 491, 574–577. 10.1038/nature11653. 23135399

[ref30] Persson B. N. J. On the theory of surface-enhanced Raman scattering. Chem. Phys. Lett. 1981, 82 (3), 561 10.1016/0009-2614(81)85441-3.

[ref31] Oren M.; Galperin M.; Nitzan A. Raman scattering from molecular conduction junctions: Charge transfer mechanism. Phys. Rev. B 2012, 85 (11), 115435 10.1103/PhysRevB.85.115435.

[ref32] Wang L.-L.; Cheng H.-P. Density functional study of the adsorption of a C_60_ monolayer on Ag(111) and Au(111) surfaces. Phys. Rev. B 2004, 69 (16), 165417 10.1103/PhysRevB.69.165417.

[ref33] Larsson J. A.; Elliott S. D.; Greer J. C.; Repp J.; Meyer G.; Allenspach A. Orientation of individual C_60_ molecules adsorbed on Cu(111): Low-temperature scanning tunneling microscopy and density functional calculations. Phys. Rev. B 2008, 77 (11), 115434 10.1103/PhysRevB.77.115434.

[ref34] Gieseking R. L. M.; Lee J.; Tallarida N.; Apkarian V. A.; Schatz G. C. Bias-Dependent Chemical Enhancement and Nonclassical Stark Effect in Tip-Enhanced Raman Spectromicroscopy of CO-Terminated Ag Tips. J. Phys. Chem. Lett. 2018, 9 (11), 3074–3080. 10.1021/acs.jpclett.8b01343. 29782171

[ref35] Braun K.; Hauler O.; Zhang D.; Wang X.; Chassé T.; Meixner A. J. Probing Bias-Induced Electron Density Shifts in Metal–Molecule Interfaces via Tip-Enhanced Raman Scattering. J. Am. Chem. Soc. 2021, 143 (4), 1816–1821. 10.1021/jacs.0c09392. 33492134

[ref36] Liu P.; Chulhai D. V.; Jensen L. Single-Molecule Imaging Using Atomistic Near-Field Tip-Enhanced Raman Spectroscopy. ACS Nano 2017, 11 (5), 5094–5102. 10.1021/acsnano.7b02058. 28463555

[ref37] Perry S. S.; Hatch S. R.; Campion A. On the role of electromagnetic field gradients in surface Raman scattering by molecules adsorbed on single crystal metal surfaces. J. Chem. Phys. 1996, 104, 6856–6859. 10.1063/1.471352.

[ref38] Yamada S.; Noda M.; Nobusada K.; Yabana K. Time-dependent density functional theory for interaction of ultrashort light pulse with thin materials. Phys. Rev. B 2018, 98 (24), 245147 10.1103/PhysRevB.98.245147.

[ref39] Galperin M.; Ratner M. A.; Nitzan A. Raman scattering in current-carrying molecular junctions. J. Chem. Phys. 2009, 130, 144109 10.1063/1.3109900. 19368431

[ref40] Galperin M.; Nitzan A. Molecular optoelectronics: the interaction of molecular conduction junctions with light. Phys. Chem. Chem. Phys. 2012, 14, 9421–9438. 10.1039/c2cp40636e. 22648067

[ref41] Roelli P.; Galland C.; Piro N.; Kippenberg T. J. Molecular cavity optomechanics as a theory of plasmon-enhanced Raman scattering. Nat. Nanotechnol. 2016, 11, 164–169. 10.1038/nnano.2015.264. 26595330

