# Supplementary material for: Charge Transfer-Mediated Dramatic Enhancement of Raman Scattering upon Molecular Point Contact Formation
Source: Nano Lett. 2022 Feb 21;22(6):2170–6. doi: 10.1021/acs.nanolett.1c02626 (PMC8949761; doi:10.1021/acs.nanolett.1c02626)
Supplement: Supplementary file 1 — nl1c02626_si_001.pdf [file nl1c02626_si_001.pdf]

Supporting Information:

Charge Transfer Mediated Dramatic Enhancement of Raman Scattering upon Molecular Point Contact Formation

Borja Cirera[1], Yair Litman[2], Chenfang Lin[1], Alaa Akkoush[2], Adnan Hammud[3], Martin Wolf[1], Mariana Rossi[2], Takashi Kumagai[1,4]*

[1]*Department of Physical Chemistry, Fritz-Haber Institute of the Max-Planck Society, Faradayweg 4-6, 14195 Berlin, Germany.*

[2]*MPI for Structure and Dynamics of Matter, Luruper Chaussee 149 22761 Hamburg, Germany*

[3]*Department of Inorganic Chemistry, Fritz-Haber Institute of the Max-Planck Society, Faradayweg 4-6, 14195 Berlin, Germany.*

[4]*Center for Mesoscopic Sciences, Institute for Molecular Science, Okazaki 444-8585, Japan*

* Corresponding author: kuma@fhi-berlin.mpg.de; kuma@ims.ac.jp

Table of Contents

1. **Materials and methods**
2. **Incident laser power dependence of the TERS intensity in the tunneling regime**
3. **Tip condition dependence of TERS for a C_{60} molecule on Ag(111)**
4. **Models used in the DFT calculations**
5. **Projected electronic density of states**
6. **Detailed analysis of the Raman active modes of C_{60} on Ag(111) and C_{60}-tip**
7. **Δz-dependent TERS of a C_{60}-tip on Ag(111) at small V_{bias}**
8. **Enlarged Δz- dependent TERS of a C_{60}–tip on Ag(111) highlighting the shifting of the vibrational modes**
9. **Simulations of the static Raman polarizability of C_{60}**
10. **Wavelength dependence of MPC-TERS**

1. Materials and methods

Sample preparation: All experiments were performed in ultra-high vacuum chambers (base pressure <5×10^{-10} mbar). The Ag(111), Au(111) and Cu(111) surfaces were purchased from MaTeck GmbH and were cleaned by repeated cycles of Ar^+ sputtering and annealing up to 670 K, 600 K and 700 K, respectively. C_{60} was purchased from Sigma-Aldrich and used without further purification. The molecules were evaporated at ~610 K from a K-cell evaporator onto the substrates held at room temperature.

STM measurement: We used a low-temperature STM from UNISOKU Ltd. (modified USM-1400) that is operated with Nanonis SPM Controller from SPECS GmbH. The bias voltage (V_{bias}) was applied to the sample, and the tip was grounded. The tunneling current (j_{STM}) was collected from the tip. We have used Ag tips fabricated by focused ion beam milling.[1]

TERS measurement: The excitation laser was focused to the STM junction with an Ag-coated parabolic mirror (numerical aperture of ~0.6) mounted on the cold STM stage. The parabolic mirror was precisely aligned using piezo motors (Attocube GmbH) that allows three translational and two rotational motions. In the Raman measurements we used a solid-state lasers for 532 nm (Cobolt) and a HeNe laser for 633 nm, with a diameter of 2 mm. This results in a beam waist <2 μm using sources in the visible range. The incident beam is linearly polarized along the tip axis (p-polarization). The scattered photons are collected by the same parabolic mirror and detected outside of the UHV chamber with a grating spectrometer (AndorShamrock 303i). The spectra presented in this work have not been background corrected or any further data treatment.

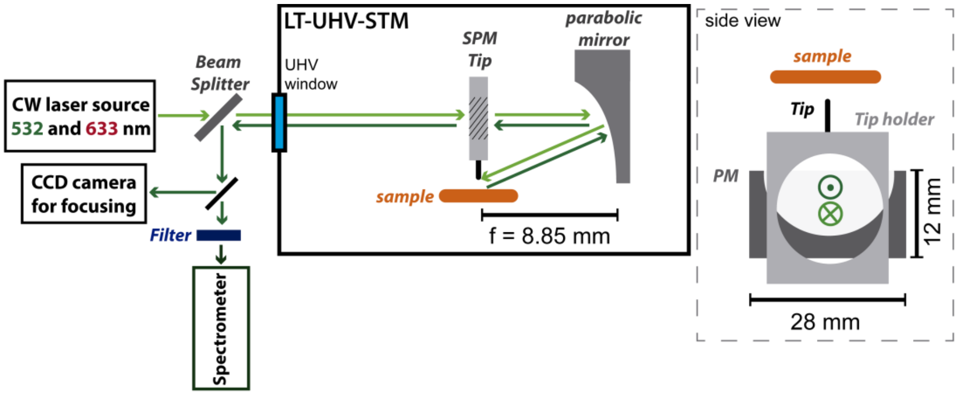

Scheme S1. Experimental set-up of the TERS measurement, with the dimensions and focal length of the parabolic mirror. The side view highlights the hollow tip holder, leaving a free path to/from the parabolic mirror (PM) for the the in- and out-coming beams.

DFT calculations: All the electronic structure calculations were carried out using FHI-aims; a numeric atom-centered orbitals, all-electron code,[2] the PBE exchange correlation functional augmented with pairwise van der Waals interactions was used.[3] When appropriate a specific modification of these corrections, aimed for surface adsorption calculations,[4] was used. “Light”

settings for numerical grids and basis sets were employed unless specified. The Ag(111) surface was represented by a (6 x 5) orthogonal surface unit cell containing three layers. A vacuum of 80 Å and a 3 x 3 x 1 *k*-point grid were used. Two pyramidal Ag tip structures were considered. The Tip A structure has five Ag layers with only one atom at the tip apex representing a total of 35 Ag atoms. Tip B was created by taking out one Ag atom from Tip A to produce a new tip with three atoms at the apex. The molecular point contact (MPC) structures were modeled using the Tip B structure, the C_{60} molecule and the Ag(111) surface. The MPC structure at equilibrium was constructed by placing the C_{60} molecule between the surface and the tip at the corresponding equilibrium adsorption distances. Compressed MPC structures were also considered by displacing the tip and the molecule towards the surface. All the geometries were relaxed keeping fixed the top two layers of the tip structures and the bottom layer of the surface. In **Fig. S3** we present all the structures models considered in this work. Harmonic frequencies of vibration were obtained through a symmetric finite-difference evaluation and subsequent diagonalization of the dynamical matrix. Atoms were displaced between 0.002 and 0.005 Å in all cartesian directions. The Raman active modes were identified from symmetry considerations and corroborated for the isolated molecule by computing the non-resonant vibrational Raman spectrum through density-functional perturbation theory calculations considering a homogeneous electric field perturbation, see **Table S1**, **S2**, and **S3**.[5] The response of electronic densities to homogeneous electric fields was calculated the isolated C_{60} molecule, the C_{60} molecule on a tip apex, the C_{60} molecule on the Ag(111) surface and for the C_{60} molecule in contact with both tip and surface, see **Fig. S4**. This quantity is directly related to the static polarizability of the molecule.[5]

**Figure**	**λ_{ext} (nm)**	**Acquisition time (s)**	**Number of spectra**	**Grating (l/mm)**	**P_{inc} (mWμm^{-2})**
1 (d)	532	15	1	1200	0.33
1 (e)	633	5	1	600	0.5
3	532	5	60	600	0.33
4 (a)	532	4	50	600	0.33
4 (b)	633	2	48	600	0.45
4 (c)	633	4	30	600	0.45
5 (b)	532	2	60	600	0.33
S1	532	4	1	600	-
S2	532	30	1	1200	0.33
S7 (a) (b) (c)	532	2	30	600	0.33

Table S1. Acquisition parameters of TERS.

2. Incident laser power dependence of TERS in the tunneling regime: C_{60} on Ag(111)

As shown in **Fig. S1**, TERS intensity of a C_{60} molecule on Ag(111) linearly depends on the incident laser power, indicating that the process is a spontaneous Raman scattering.

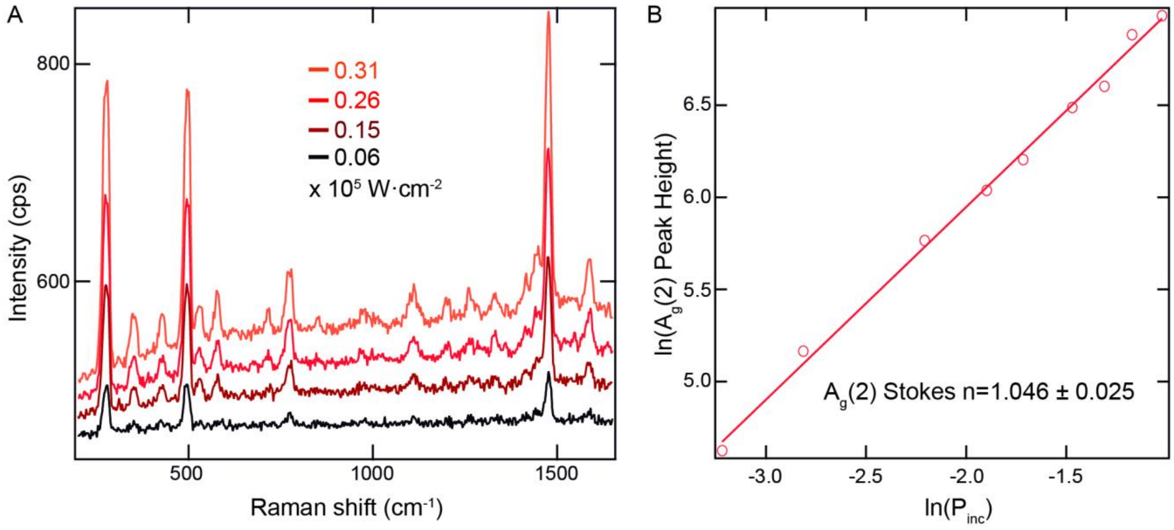

Figure S1. (a) Stokes Raman spectra at four different I_{inc}, under 532 nm illumination of a C_{60} film on Ag(111), measured with a clean Ag Tip (Initial set point: V_{bias}=50 mV, j_{STM} =2 nA, P_{inc} = 0.31×10^5 W cm^{-2}). **(b)** Log plot of Stokes intensity of $A_g(2)$ vs. P_{inc}, showing a power dependence with n=1.

3. Tip condition dependence of TERS for a C_{60} molecule on Ag(111)

We found that the TERS intensity depends on the tip conditions. **Figure S2** shows the TER spectra recorded for a C_{60} molecule on Ag(111) under different tip conditions modified by poking the tip apex into the bare surface in a controlled manner. This procedure will change the atomic-scale structure of the tip apex. The change of the apex structure may be reflected in different spectral fingerprint at <200 cm^{-1} which can be assigned to the phonons of the Ag tip. It is likely that the different atomistic structures at the apex have a significant impact on the field enhancement and distribution in the STM junction.[6, 7]

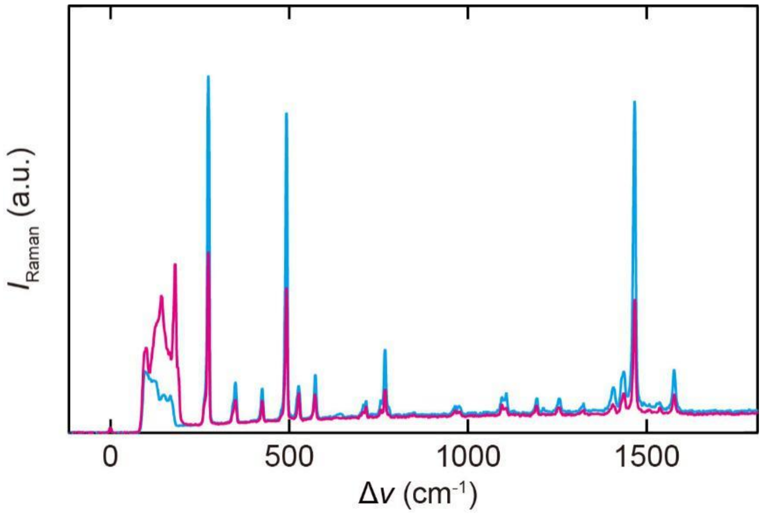

Figure S2. TER spectra recorded for a C_{60} molecule on the Ag(111) surface under different tip conditions (V_{bias}=0.1 V, j_{STM}=2.5 nA, λ_{ext}=532 nm, P_{inc}=0.33×10^5 W cm^{-2}, t_{exp}=30 s). The intensity is normalized by the Rayleigh line.

4. Models of C_{60}-tip used in the DFT calculations

Figure S3 shows the different structural models used for the DFT simulations.

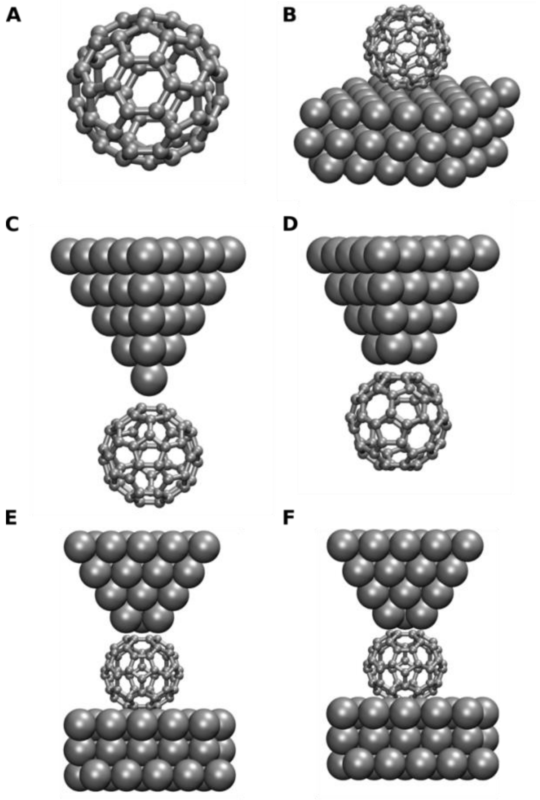

Figure S3. (a) Isolated C_{60} molecule. **(b)** C_{60} adsorbed on the Ag(111) surface. **(c)** Fused C_{60} tip with one atom at the tip apex (Tip A). **(d)** Fused C_{60} tip with three atoms at the tip apex (Tip B). **(e)** MPC structure at equilibrium. **(f)** MPC structure compressed by 1.5 Å.

5. Projected electronic density of states

Projected electronic density of states (PDOS) were simulated for the different structures in **Fig. S3** and are presented in **Fig. S4**. Additional calculations with the HSE06 exchange correlation functional[8] were performed and confirmed the results obtained with the PBE exchange correlation functional (see **Fig. S5**). Moreover, the LUMO of the C_{60} molecule is in resonance with the Fermi-level of the system, a phenomenon normally referred to as Fermi-level pinning. In these situations, the lack of band-gap renormalization upon adsorption 'cancels out' with the band-gap underestimation by the PBE functional and leads to a fortuitous cancellation of errors. As a result, it is obtained a better description of the orbitals than might be expected otherwise

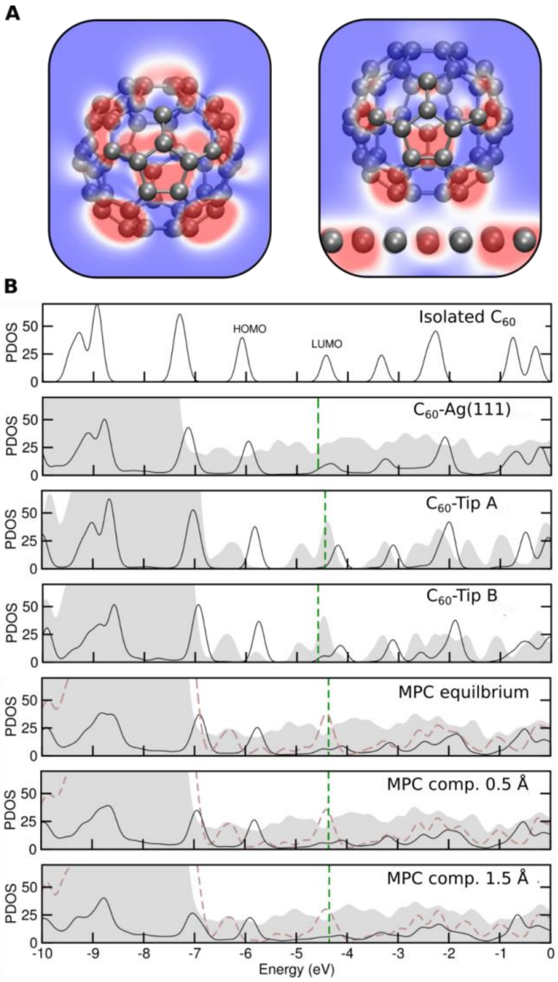

Figure S4. (**a**) Two-dimensional cuts of the spatial distribution of electronic density corresponding to the LUMO of isolated C_{60} molecule (left) and of the C_{60} molecule adsorbed on the Ag(111) surface (right). **(b)** Atom-projected electronic density of states (PDOS) for the different configurations (see **Fig. S3**). C atoms, Ag surface atoms and Ag tip atoms are depicted with solid black line, gray area, and dashed brown line, respectively. The Fermi energy is marked by a vertical green dashed line in each panel.

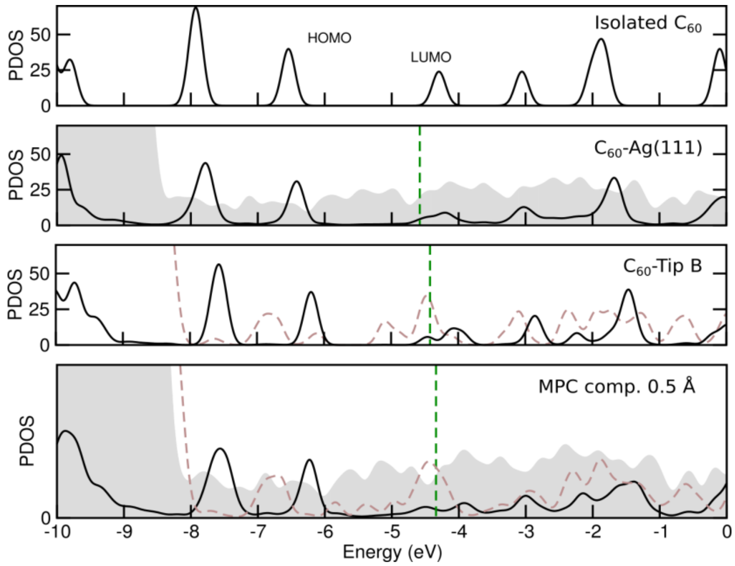

Figure S5. Same as **Fig. S4** using the HSE06 exchange correlation functional.

6. Detailed analysis of the Raman active modes of C_{60} on Ag(111) and C_{60}-tip

According to group theory, the normal modes belong to the following representations

$$\Gamma = 2A_g + 3F_{1g} + 4F_{2g} + 6G_g + 8H_g + A_u + 4F_{1u} + 5F_{2u} + 6G_u + 7H_u,$$

where subscripts g (gerade) and u (ungrade) represent the symmetry of the eigenvector under the action of the inversion operator and the symmetry labels refer to irreducible representations.[9] The icosahedral symmetry yields 10 distinct Raman-active modes ($2A_g$+$8H_g$) and 4 infrared active modes ($4F_{1u}$) up to first order. Calculated vibrational harmonic frequencies for the gas-phase C_{60} and C_{60} on Ag(111) are reported in **Table S1** (red background), in good agreement with the experimentally observed values (blue background). The comparison with the experimentally obtained frequencies in solid state (ref. 21 in the main text) offers information regarding the nature of the observed shifts. It can be seen that the coupling of the C_{60} molecule with Ag atoms breaks the 5-fold degeneracy of the H_g modes while the A_g modes do not present appreciable mixing (see also **Table S2**). The third column describes the calculated frequencies for the C_{60} monomer relaxed geometry upon adsorption on Ag(111), without taking into account the change in the electronic structure, by removing the substrate after relaxing the geometry. The forth column contains additionally the contribution of the surface, taking into account hybridization (electronic charge rearrangement). In all cases the vibrational frequencies are red-shifted in comparison to the isolated molecule showing an overall softening of the vibrational modes.

**Mode**	**Solid state** (Ref. 21)	**Gas Phase (Sim.)**	**C_{60}-Ag(111) only Relaxed (Sim.)**	**C_{60}-Ag(111) relaxed+CT (Sim.)**	**C_{60} in an island (Exp.)**
H_g(8)	1577.5	1565 (5)	1552-1559	1530-1550	1576.4 ± 0.2
A_g(2)	1470.0	1477 (1)	1464	1461	1466.1 ± 0.1
H_g(7)	1426.5	1426 (5)	1423	1377-1417	1435.4 ± 0.3
H_g(6)	1251.0	1248 (5)	1238-1249	1240-1244	1255.5 ± 0.5
H_g(5)	1101.0	1104 (5)	1099-1105	1082-1103	1103.0 ± 0.7
H_g(4)	775.0	775 (5)	769-773	763-769	768.1 ± 0.1
H_g(3)	711.0	702 (5)	703-704	621-699	711.6 ± 0.7
A_g(1)	497.5	491 (1)	487	488	492.4 ± 0.02
H_g(2)	432.5	424 (5)	425-428	405-416	423.3 ± 0.4
H_g(1)	273.0	258 (5)	258-259	255-266	273.8 ± 0.02

Table S2. Theoretical (red) and experimental (blue) vibrational harmonic frequencies. Only the modes that are Raman active in gas phase are presented. Numbers in parenthesis show the degeneracy of each mode in the gas phase and frequency intervals indicate the extent in which the degeneracy of the modes is broken. All mode frequencies in cm^{-1}.

In **Table S3**, the calculated vibrational frequencies of C_{60} adsorbed on the tips with slightly different geometries (Tip A and B in **Fig. S3**) are presented. The first column show results that only account for the effect of the relaxed geometry (deformation) of the C_{60} molecule adsorbed on Tip A, while column 2 also includes the effect of the electron transfer from Tip A. Similar data is presented in columns 3 and 4 for Tip B. The effect of mechanical deformation of an isolated C_{60} molecule is presented (C_{60} hexagon compressed) in the fifth column, in order to estimate the effect of such a deformation in the fused junctions. The molecule was deformed by bringing the six C atoms of opposing hexagonal rings of the molecule closer by 0.2 Å. These positions were fixed and all other degrees of freedom of the molecule were relaxed. In column 6, the frequencies calculated for the contact structure with Tip B are shown. The calculated frequencies of C_{60} upon adsorption on the tip show small differences for subtle variations of the tip apex structure, resulting from slightly different adsorption geometries of C_{60}, which eventually affect the electronic charge rearrangement between the molecule and the tip. This will account for the experimentally observed variations between different tips (**Table S3**).

**Frequency (cm^{-1})**	**Mode decomposition**
**1550**	H_g(8) (36 %) + H_u (60 %)
1550	H_g(8) (22 %) + H_u (56 %)
1547	H_g(8)
1546	H_g(8) (46 %) + H_u (47 %)
1546	H_g(8) (45 %) + H_u (47 %)
1530	H_g(8) (52 %) + H_u (40 %)
**1530**	H_g(8) (56 %) + H_u (37 %)
1461	A_g(2)
1417	H_g(7) (15 %) + T_{1u} (50 %) + G_{1u} (17 %)
1396	H_g(7)
1396	H_g(7) (55 %) + T_{1u} (26 %)
1395	H_g(7)
1378	H_g(7)
1377	H_g(7)
**1244**	H_g(6)
1244	H_g(6)
1243	H_g(6)
1240	H_g(6)
**1240**	H_g(6)
**1103**	H_g(5)
1100	H_g(5)
1094	H_g(5) (40 %)+G_g (50 %)
1094	H_g(5) (44 %)+G_g (52 %)
1094	H_g(5) (22 %)+G_g (72 %)
1094	H_g(5)
1094	H_g(5) (36 %)+G_g (63 %)
1083	H_g(5)
**1082**	H_g(5)
**769**	H_g(4)
769	H_g(4)
767	H_g(4)
766	H_g(4)
**763**	H_g(4)
**488**	A_g(1)
699	H_g(3) (26 %) + T_{3u}(2) (68 %)
699	H_g(3) (35 %) + T_{3u}(2) (64 %)
699	H_g(3) (37 %) + T_{3u}(2) (56 %)
689	H_g(3) (69 %) + T_{3u}(2) (26 %)
681	H_g(3)
681	H_g(3)

621	H_g(3) (33 %) + T_{3u}(2) (13 %)
**416**	H_g(2)
411	H_g(2)
411	H_g(2)
405	H_g(2)
**405**	H_g(2) (75 %)
**266**	H_g(1)
258	H_g(1)
258	H_g(1)
255	H_g(1)
**255**	H_g(1)

Table S3. Normal modes decomposition for Raman active modes in the C_{60} on Ag(111). This decomposition was obtained by a projection of the normal modes calculated for the surface-adsorbed molecule on the modes calculated for the isolated molecule, which have the labeled symmetries. Only modes that contribute more than 10 % to a particular isolated-molecule mode are reported. Numbers in bold indicate the limit of the intervals reported in **Table S2**. Similar mixing is observed for the C_{60} on Tip B and C_{60} on Tip A (not shown).

**Mode**	**C_{60}–Tip A (only relaxed)**	**C_{60}–Tip A (relaxed + charge trans.**	**C_{60}–Tip B (only relaxed)**	**C_{60}–Tip B (relaxed .+ charge trans.)**	**C_{60} hex. compress ed**	**C_{60}–Tip B on Ag(111) contact equilibrium**	**Ag tip #1 on Ag(111)**		**Ag tip #2 on Ag(111)**		**Ag tip #3 on Ag(111)**	
							tunneling	MPC	tunneling	MPC	tunneling	MPC
***H_g*(8)**	1560-1566	1535-1561	1555-1563	1536-1557	1566-1580	1507-1537	1572.7 ± 1.4	1537.9± 0.8	1594.2 ± 1.1	1586.6 ± 0.7	1590.0 ± 0.5	1587.4 ± 0.7
***A_g*(2)**	1474	1471	1470	1465	1486	1447	1465.4 ± 0.1	1459.8 ± 0.1	1477.5 ± 0.2	1468.4 ± 0.2	1479.0 ± 0.2	1469.7 ± 0.3
***H_g*(7)**	1403	1370-1410	1368	1370-1400	1427	1366-1384	1424.2 ± 1.0	1410.7 ± 0.5	1414.7 ± 1.1	1411.3 ± 0.4	1455.9 ± 1.8	1414.2 ± 0.2
***H_g*(6)**	1241-1251	1244-1247	1239-1253	1236-1246	1242-1264	1231-1245	1260.0 ± 2.2	1262.0 ± 1.2	N.A.	1264.5 ± 1.6	1267.1 ± 0.9	1262.8 ± 1.2
***H_g*(5)**	1100-1106	1096-1103	1098-1106	1081-1104	1109-1110	1070-1092	1105.7 ± 1.5	1110.0 ± 2.3	1110.6 ± 1.4	1104.0 ± 0.4	1105.3 ± 0.8	1102.2 ± 0.9
***H_g*(4)**	773-776	770-774	768-775	763-772	770-783	753-767	773.6 ± 1.4	764.1 ± 0.6	773.8 ± 0.8	768.4 ± 0.3	775.7 ± 0.4	768.4 ± 0.3
***H_g*(3)**	702-703	670-695	702-703	688-701	700-703	624-674	712.5 ± 3.0	704.8 ± 3.1	726.4 ± 3.0	723.6 ± 13.6	702.8 ± 3.8	704.0 ± 0.6
***A_g*(1)**	490	490	489	488	494	489	488.4 ± 0.2	488.0 ± 0.1	491.6 ± 0.3	488.4 ± 0.1	492.4 ± 0.2	487.0 ± 0.2
***H_g*(2)**	424-426	416-420	424-427	399-424	419-427	395-399	412.6 ± 0.9)	411.6 ± 1.2	428.4 ± 1.5	417.4 ± 0.3	420.4 ± 0.5	421.3 ± 0.2
***H_g*(1)**	258-259	258-263	258-259	253-256	247-268	255-259	266.6 ± 0.6)	273.3 ± 0.1	270 ± 0.3	268.3 ± 0.1	266.2 ± 1.3	269.1 ± 0.1

Table S4. Calculated (red columns) and experimental (blue columns) frequencies of C_{60} vibrations for different contact situations (calculation) and for different tips (experiment). Only the modes which are Raman active in the gas phase are presented. Units in cm^{-1}.

7. Δz- dependent TERS of a C_{60}-tip on Ag(111) at small V_{bias}

Various Δz-dependent TERS measured with increasing V_{bias} show that at this regime the TERS intensity at is not sensitive to changes in the direct current in the STM.

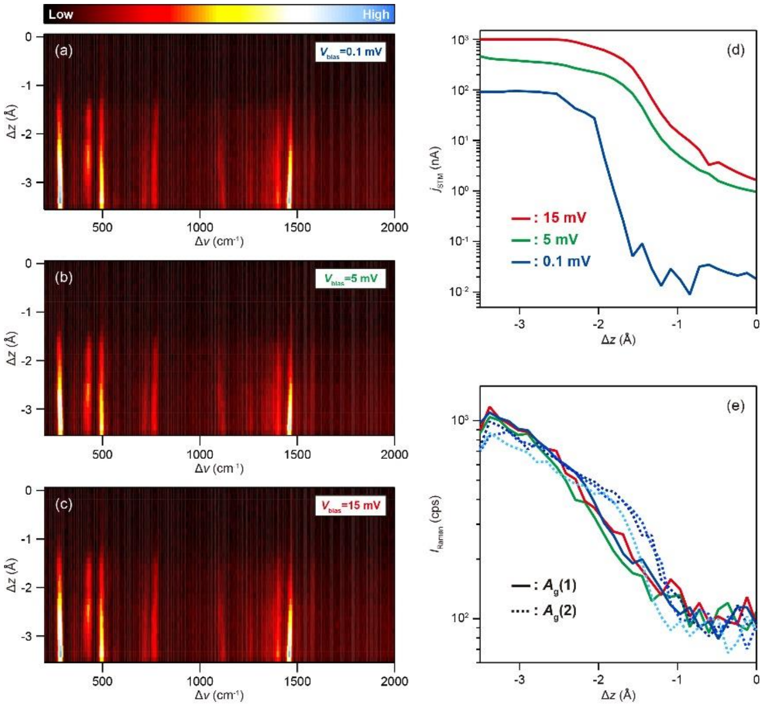

Figure S6. (a–c) Δz-dependent TER spectra of a C_{60}-tip approaching the Ag(111) surface at 0.1, 5 and 15 mV, respectively (λ_{ext}=532 nm, P_{inc}=0.33 mWμm^{-2}, 10 K). (d) Simultaneously obtained j_{STM}–Δz curves for the three different cases, with j_{STM} at contact differing by one order of magnitude. (e) Intensity of the $A_g(1)$ and $A_g(2)$ modes as a function of the Δz for the different V_{bias} (j_{STM}), showing almost no differences.

8. Enlarged Δz-dependent TERS of a C_{60}–tip on Ag(111) highlighting shifting of the vibrational modes

Figure S7 show the enlarged Δz-dependent TERS of a C_{60}–tip on Ag(111) in **Fig. 2c**.

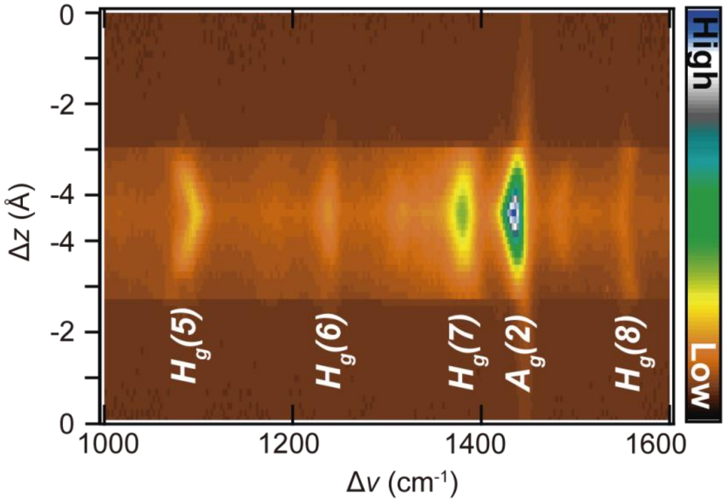

Figure S7. Enlarged Δz-dependent TERS of **Fig. 3** (λ_{ext}=532 nm, P_{inc}=0.33 mWμm^{-2}, 10 K).

9. Simulations of the non-resonant Raman intensity of in the various environments

The *zz* component of the polarizability tensor of the systems was calculated through the explicit application of a homogeneous electric field in the *z* direction (perpendicular to the surface; see orientation of all systems in **Fig. S3**), with a subsequent finite-difference evaluation of the derivative of the dipole moment in the *z* direction. We applied fields of strengths 10^{-4} to 10^{-3} V/Å and converged densities down to 10^{-8} bohr^{-3}, in addition to employing *tight* settings of the FHI-aims code for these calculations. We then calculated the Raman intensities through finite differences of the *zz* component of the polarizability tensor with respect to the displacements along the normal modes. All simulations were performed with the PBE density functional and all finite difference derivatives were computed by central differences. We assessed that a possible numerical error of up to 20 % can be present in the numbers reported. We found that lateral interactions in the periodic simulations could strongly affect the calculated intensities. The overall effect of such interaction is a relative decrease of the derivative of the zz component of the polarizability tensor with respect to the isolated C_{60} value (see **Tables S5** and **S6**). By comparing to isolated calculations of the systems that do not contain the Ag(111) surface, we concluded that unit cells with at least 40 Å of length along the lateral distances were necessary to obtain the numbers reported in **Table S5** which are minimally affected by the spurious lateral interaction (see **Fig. S8**).

Mode	Isolated C_{60}	C_{60}-Ag(111)	C_{60}-Tip A	C_{60}-Tip B	MPC at equilibrium
$A_g(1)$	1.0	1.6	8.0	1.9	0.1
$A_g(2)$	1.0	4.7	33.1	7.7	0.2

Table S5. Derivative of the *zz* component of the polarizability tensor. The *z* axis is parallel to the surface normal and the derivatives were computed with respect to the displacements along $A_g(1)$ and $A_g(2)$ normal modes. Values are reported using the gas phase value as reference. A 46.981 Å x 40.687 Å x 104.795 Å simulation box was employed.

Mode	Isolated C_{60}	C_{60}-Ag(111)	C_{60}-Tip A	C_{60}-Tip B	MPC at equilibrium
$A_g(1)$	1.0	0.6	1.3	0.6	0.03
$A_g(2)$	1.0	2.1	6.3	2.2	0.06

Table S6. Same as **Table S5** for 14.681 Å x 15.251Å x 104.795 Å simulation box.

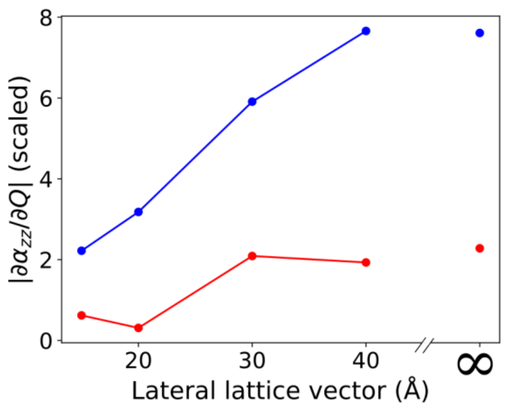

Figure S8. Derivative of the *zz* component of the polarizability tensor ($\frac{\partial\alpha}{\partial Q}$) with respect to $A_g(1)$ (red) and $A_g(2)$ (blue) normal modes as a function of the lateral lattice vectors magnitude obtained for the C_{60}-Tip B model. Infinite lateral lattice vectors refers to calculations performed without periodic boundary conditions.

10. Wavelength dependence of MPC-TERS

Figure S9 shows the waterfall plot of the TERS spectra recorded for Ag(111) and Au(111) at the excitation wavelength of 633 nm. For both surfaces, the strong Raman enhancement can be observed in the MPC regime. The enhancement factors are estimated to be $\rho_{\mathrm{MPC,\ Ag(111)}}$~32.1 and $\rho_{\mathrm{MPC,\ Au(111)}}$~40.9.

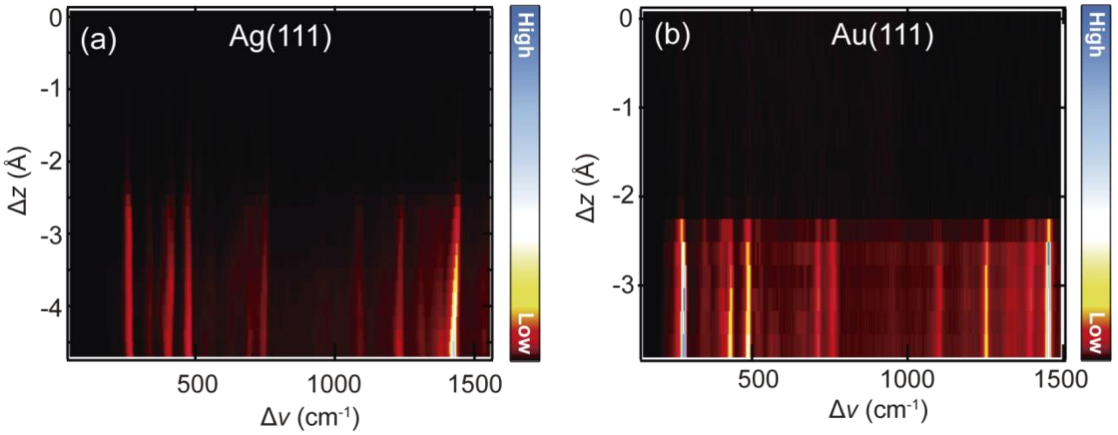

Figure S9. (a–b) Δ*z*-dependent TERS spectra measured on the Ag(111) and Au(111) surface with λ_{ext}=633 nm (Ag(111): λ_{ext}=633 nm, P_{inc}=0.45 mWμm^{-2}, V_{bias} = 0 V, 10 K, Au(111): λ_{ext}=633 nm, P_{inc}=0.45 mWμm^{-2}, V_{bias} = 0 V, 10 K).

References

[1] Böckmann, H. *et al.* Near-Field Manipulation in a Scanning Tunneling Microscope Junction with Plasmonic Fabry-Pérot Tips. *Nano Lett.* **2019**, *19*, 3597.

[2] Blum, V. *et al*. *Ab initio* molecular simulations with numeric atom-centered orbitals. *Comp. Phys. Commun.* **2009**, *180*, 21752196.

[3] Tkatchenko, A.; Scheffler, M. Accurate Molecular Van Der Waals Interactions from Ground-State Electron Density and Free-Atom Reference Data. *Phys. Rev. Lett.* **2009**, *102*, 073005.

[4] Ruiz, V. G.; Liu, W.; Zojer, E.; Scheffler, M.; Tkatchenko, A. Density-Functional Theory with Screened van der Waals Interactions for the Modeling of Hybrid Inorganic-Organic Systems. *Phys. Rev. Lett.* **2012**, *108*, 146103.

[5] Shang, H.; Raimbault, N.; Rinke, P.; Scheffler, M.; Rossi, M.; Carbogno, C. All-electron, real-space perturbation theory for homogeneous electric fields: theory, implementation, and application within DFT. *New J. Phys.* **2018**, *20*, 073040.

[6] Zhang, P.; Feist, J.; Rubio, A.; García-González, P.; García-Vidal, F. J. *Ab initio* nanoplasmonics: The impact of atomic structure. *Phys. Rev.* B **2014**, *90*, 161407(R).

[7] Urbieta, M. *et al.* Atomic-Scale Lightning Rod Effect in Plasmonic Picocavities: A Classical View to a Quantum Effect. *ACS Nano* **2018**, *12*, 585.

[8] Krukau, A. V. *et al*. Influence of the exchange screening parameter on the performance of screened hybrid functionals, *J. Chem. Phys.* **2006**, *125*, 224106.

[9] Dresselhaus, M. S.; Dresselhaus, G.; Eklund, P. C. Raman Scattering in Fullerenes. *J. Raman Spectrosc.* **1996**, *27*, 351.
